# Characterisation of *Caenorhabditis elegans* sperm transcriptome and proteome

**DOI:** 10.1186/1471-2164-15-168

**Published:** 2014-02-28

**Authors:** Xuan Ma, Yingjie Zhu, Chunfang Li, Peng Xue, Yanmei Zhao, Shilin Chen, Fuquan Yang, Long Miao

**Affiliations:** 1 Laboratory of Non-coding RNA, Institute of Biophysics, Chinese Academy of Sciences, Beijing 100101, China; 2 Institute of Medicinal Plant Development, Chinese Academy of Medical Sciences, Beijing 100094, China; 3 Key Laboratory of Protein and Peptide Pharmaceuticals & Laboratory of Proteomics, Institute of Biophysics, Chinese Academy of Sciences, Beijing 100101, China

**Keywords:** *C. elegans*, Sperm, Transcriptome, Proteome

## Abstract

**Background:**

Although sperm is transcriptionally and translationally quiescent, complex populations of RNAs, including mRNAs and non-coding RNAs, exist in sperm. Previous microarray analysis of germ cell mutants identified hundreds of sperm genes in *Caenorhabditis elegans*. To take a more comprehensive view on *C. elegans* sperm genes, here, we isolate highly pure sperm cells and employ high-throughput technologies to obtain sperm transcriptome and proteome.

**Results:**

First, sperm transcriptome consists of considerable amounts of non-coding RNAs, many of which have not been annotated and may play functional roles during spermatogenesis. Second, apart from kinases/phosphatases as previously reported, ion binding proteins are also enriched in sperm, underlying the crucial roles of intracellular ions in post-translational regulation in sperm. Third, while the majority of sperm genes/proteins have low abundance, a small number of sperm genes/proteins are hugely enriched in sperm, implying that sperm only rely on a small set of proteins for post-translational regulation. Lastly, by extensive RNAi screening of sperm enriched genes, we identified a few genes that control fertility. Our further analysis reveals a tight correlation between sperm transcriptome and sperm small RNAome, suggesting that the endogenous siRNAs strongly repress sperm genes. This leads to an idea that the inefficient RNAi screening of sperm genes, a phenomenon currently with unknown causes, might result from the competition between the endogenous RNAi pathway and the exogenous RNAi pathway.

**Conclusions:**

Together, the obtained sperm transcriptome and proteome serve as valuable resources to systematically study spermatogenesis in *C. elegans*.

## Background

Spermatogenesis is a process during which undifferentiated germ cells develop into mature sperm cells. A sperm cell has a highly condensed nucleus and lacks many organelles, such as ribosomes and Golgi apparatus; thus, sperm is transcriptionally and translationally quiescent [1-7]. Early observations that RNAs exist in sperm were originally dismissed [8]; however, over the past decade, the view that sperm contains complex RNA populations, including mRNAs and non-coding RNAs, has become established [1-7]. These RNA populations contain not only non-functional remnant spermatogenesis-expressed transcripts, but also RNAs with potential functions during early embryogenesis [9-11]. Recently, the rapid development of microarray and high-throughput sequencing technologies has assisted the profiling of sperm RNAs in a broad range of species, including human [12,13], mouse [13], invertebrates [14-17] and plants [18-22].

The germline of *Caenorhabditis elegans* is transparent, and is well suited for cellular characterization of spermatogenesis [23]. The transcription of sperm genes is initiated during the pachytene stage; when spermatocytes enter the karyosome stage, global transcription ceases [24-26]. In *C. elegans*, approximately 60 genes that are essential for normal spermatogenesis have been characterized [23,27]. By microarray analyses, Reinke *et al.* identified sets of genes that are spermatogenesis-enriched, oogenesis-enriched and sex-regulated [28,29]. Notably, their analyses showed that the sperm-enriched genes encode considerable numbers of kinases and phosphatases, and are depleted from the X-chromosome, echoing the findings of Reuben *et al.* that the X-chromosome in males exhibits striking H3K9 methylation [30]. These microarray analyses identified sperm genes on a large-scale; however, the analyses were based on comparisons between germ cell mutants, and purified sperm cells were not used. In addition, their microarray analyses only identified sperm-enriched genes, while omitted those sperm genes that are also abundant in oocyte.

Compared with sperm genes, the *C. elegans* sperm small RNAs have been investigated more extensively. A few studies reported deep sequencing of the small interfering RNAs (siRNAs) of spermatogenic cells [14-17]. It was demonstrated that the ERI-1/RRF-3/ALG-3 pathway is essential for production of the spermatogenesis-specific 26G endogenous siRNAs (endo-siRNAs) [17]. Han *et al.* also showed that 26G endo-siRNAs regulate spermatogenic gene expressions [15].

In the present study, we purified highly pure *C. elegans* sperm cells and applied high-throughput approaches to obtain sperm transcriptome and proteome. A large number of long or intermediate-sized non-coding RNAs (lncRNAs hereafter) are found in sperm transcriptome, implying the important roles of lncRNAs during spermatogenesis. We showed that sperm proteome/transcriptome is enriched in not only kinase and phosphatase proteins/genes as previously reported [28,29], but also ion binding proteins/corresponding genes, underlying the crucial roles of intracellular ions in sperm. Our extensive RNAi screening of sperm genes did not produce many defective phenotypes. Our further analysis revealed a tight correlation between sperm transcriptome and sperm small RNAome, which leads to a view that the phenomenon of inefficient RNAi screening might result from the endogenous RNAi pahway that acts strongly during spermatogenesis and is to compete over the exogenous RNAi pathway.

## Results

### Sequencing *C. elegans* sperm transcriptome and proteome

**Figure 1:**
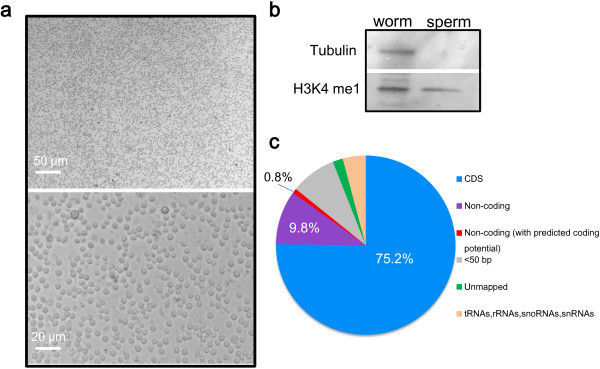
**Sequencing of *C. elegans *sperm transcriptome. (a)** Microscopic image of purified sperm cells. **(b)** Whole worms and concentrated sperm were analyzed by immunoblotting using antibodies against mono-methylated H3K4 and tubulin. No tubulin was detected in the sperm suggesting high purity of the prepared sample. **(c)**  Classification of the sperm transcriptome sequencing reads. Most of the reads are coding sequences (CDS); about ~10% of the reads are non-coding sequences including those with predicted coding potential. Reads shorter than 50 bp, unmapped to the *C. elegans* genome or coding tRNAs, rRNAs, snoRNAs and snRNAs were filtered out before assembly.

We performed large-scale culturing of *C. elegans* strain *him-5*, followed by isolating males and purifying sperm, and obtained highly pure sperm cells. These cells contain nearly no residue bodies or primary spermatocytes, as examined by microscope (Figure 1a). To further examine the purity of acquired sperm, these concentrated sperm as well as the whole worms were subjected to Western blot analysis using antibodies against tubulin and H3K4 mono-methylation mark. Trace H3K4 methylation mark has been detected in mammalian sperm [1,5,7]; here, we show *C. elegans* sperm retain histone methylation mark (Figure 1b). In contrast, tubulin which is not transmitted to mature sperm is not detected in our prepared sperm (Figure 1b). This suggests the purified sperm cells were of high purity.

**Figure 2:**
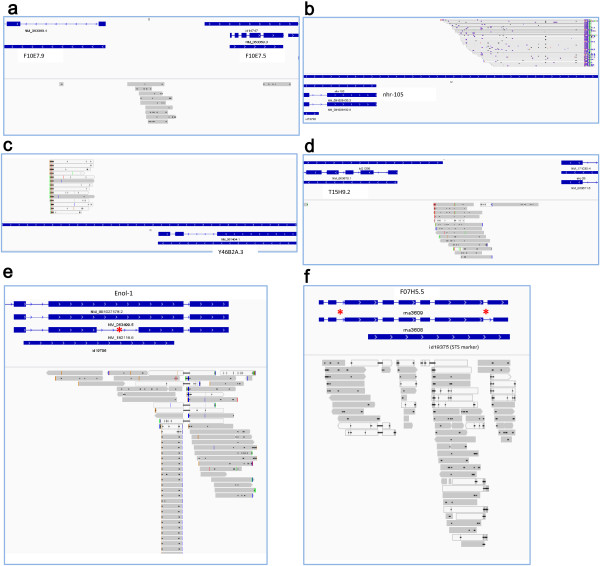
**Novel non-coding RNA transcripts and gene splicing isoforms. (a)**, **(b)**, **(c)** and **(d)** illustrate new non-coding RNA transcripts supported by sequencing reads. **(e)** and **(f)** show new gene splicing variants. Red asterisks point to the introns covered by reads.

RNA was extracted from these cells, followed by cDNA synthesis and amplification, and the purified cDNA was deep sequenced on a 454 GS FLX Titanium platform. High-throughput sequencing produced 367,638 high quality (HQ) reads with an average length of 315 base-pairs (bp) (Additional file 1: Figure S1 and Table S1). These HQ sequences were filtered to remove repeat, tRNAs, rRNA, snoRNA and snRNA sequences (see Methods), and then assembled by Newbler (version 2.3) using the *C. elegans* coding sequence dataset (WS228) as a reference, and 9,287 coding transcripts were identified (Additional file 2). The sequences unmapped to the *C. elegans* coding transcriptome were further assembled using the *C. elegans* genome as a reference to identify possible non-coding transcripts (Additional file 3). In summary, the majority of the sequences (75.2%) are coding transcripts, and 10.6% of the sequences were identified as non-coding transcripts (Figure 1c). A small part of these non-coding sequences (0.8%) are predicted to have coding potential by CPAT (version 1.2.1) (Additional file 3). In the non-coding portion, many novel transcripts supported by overlapping reads were identified (Figure 2a-d, also see next section). Meanwhile, although the sequencing depth is limited, we did find a few new splicing isoforms, *e.g.*, the asterisks in Figure 2e,f point to the introns covered by reads indicating the introns can be transcribed.

We further assessed the level of possible contaminations for sperm transcriptome by examining 15 somatic marker genes (Additional file 1: Table S2) for their abundances in the trancriptome dataset. Only five (*wrt-2*, *ges-1*, *hlh-17*, *myo-2* and *myo-3*) were detected in sperm transcriptome, and the number of reads for these five genes ranged only from 1 to 3, thus, somatic cell contamination is much low in the prepared sperm cells. The RPKM (Reads Per Kilobase of exon model per Million mapped reads) values for these five genes range from 3.42e-4 to 3.27e-3. To obtain a higher confidence transcriptome dataset, we set the RPKM cut-off to 3.27e-3. The resultant sperm transcriptome dataset contains 3,760 genes (Additional file 4) and our following analyses are based on these 3,760 genes.

In parallel with sperm mRNA sequencing, sperm protein extract was prepared and separated on SDS-polyacrylamide gel prior to removal of the MSP fraction. Subsequently, the protein extract was processed for shotgun LC-MS/MS analysis. LC-MS/MS analysis identified 27,667 peptides, corresponding to 2,994 proteins in the *C. elegans* protein dataset (WS229), with a probability >95% using percolator scores (data available from http://159.226.118.206/miaolab/C.elegans%20data.htm). None of the above-mentioned somatic cell markers were detected in this proteome dataset. 207 proteins (6.9%) were degenerative (*i.e.*, they cannot be distinguished among homologous proteins).

**Figure 3:**
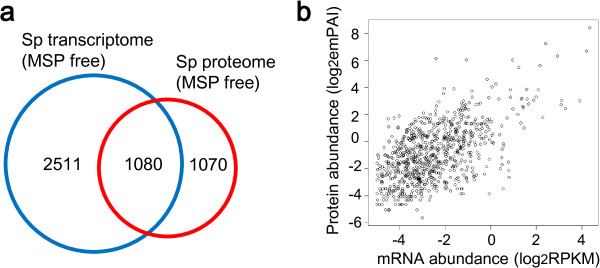
**Comparison of sperm transcriptome and proteome. (a)** Venn diagram showing the overlap between the sperm transcriptome and the sperm proteome (MSPs and predicted proteins were excluded). The number of genes/proteins in each part is indicated. **(b)** Plot showing sperm mRNA and protein abundances are well correlated.

Next, we compared sperm transcriptome and proteome (MSPs and the predicted proteins were excluded). The result showed that 30.1% of the transcriptome overlaps with 50.2% of the proteome (Figure 3a and Additional file 5). The limited overlap between the two–omes might suggest the different compositions of sperm transcriptome and proteome. Alternatively, it may results from the insufficient sequencing depth. When using RPKM and emPAI (exponentially modified protein abundance index) values to evaluate mRNA and protein abundances, we found that in the overlapping portion, mRNA and protein abundances are significantly correlated (*r* = 0.58, *P* < 2.2e-16, Pearson test) (Figure 3b). The overlapping portion contains the most abundant non-MSP sperm genes/proteins.

### Validation of novel lncRNAs

**Figure 4:**
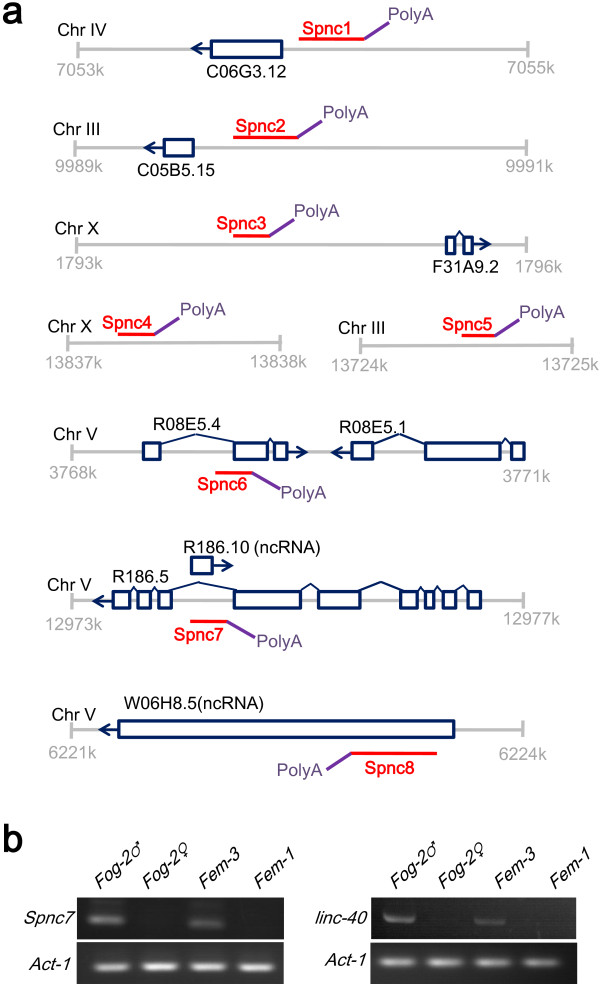
**Identification of transcripts of novel lncRNAs. (a)** Schematic representation of the 3′ structures of eight novel lncRNAs, namely *Spnc1-8* (in red), and their poly(A) tails (in purple), which were confirmed by 3′RACE. Known gene models are shown in dark blue. **(b)** Identification of the male-specific lncRNA *Spnc7* and long non-coding RNA *linc-40* by RT-PCR. The *Act-1* gene was used as control.

As mentioned above, sperm transcriptome comprises ~10% of non-coding RNAs. It has been proposed that over 26% and 13% of the full-length cDNAs in human and mice, respectively, are mRNA-like ncRNAs [31-33]. In *Drosophila*, some lncRNAs are thought to be important for male-specific processes [34]. Therefore, we selected 30 lncRNA candidates that have poly(A/T) tail from sperm transcriptome and verified them by 3′RACE. Eight novel lncRNAs (*Spnc1*-*8*) were successfully validated by 3′RACE (Figure 4a and Additional file 6), and *Spnc7* was expressed specifically in males (Figure 4b, note that at 25°C, the mutant *fem-3* overproduces sperm while *fem-1* only produces oocytes; *fog-2* generates separate male and female offspring).

Recently, Nam *et al.* identified ~170 polyadenylated lncRNAs in *C. elegans*, and many of these lncRNAs are predicted to be associated with male identity and sperm functions [35]. Thus, we compared these lncRNAs with the non-coding portion of our sperm transcriptome dataset by BLAST (*E*-value = 9e-13, this *E*-value cutoff allows alignment to have ~60 bp contiguous perfect match). 32 lncRNAs were found in sperm transcriptome, and *linc-40* was shown to be male-specific (Figure 4b). Together, these results suggest that apart from coding genes, substantial numbers of lncRNAs are expressed and may play functional roles during spermatogenesis.

### Sperm are enriched in ion binding proteins

**Figure 5:**
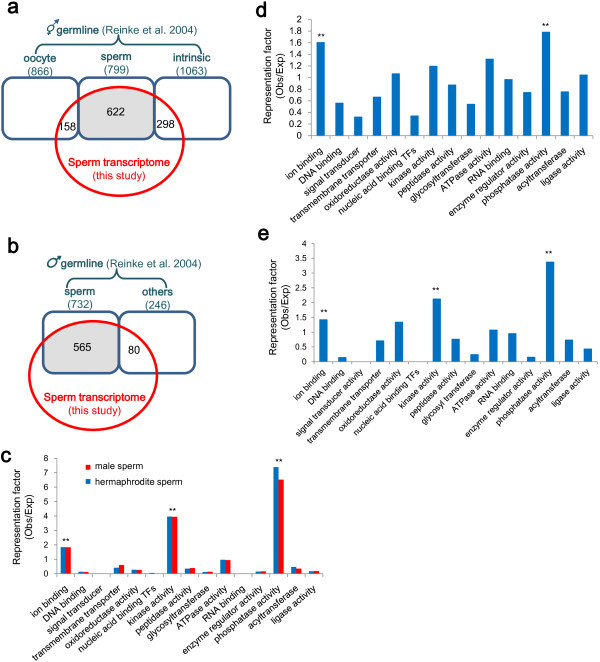
**Comparison of the sperm transcriptome with the available microarray datasets. (a)** Venn diagram showing the overlap between the sperm transcriptome (in red) and the hermaphrodite germline gene datasets obtained by microarray analysis (in blue). The total number of genes in each dataset and the number of genes in the overlapping portion are indicated. **(b)** Venn diagram showing the overlap between the sperm transcriptome (in red) and the male germline gene datasets (in blue). **(c)** GO analysis of the male and hermaphrodite spermatogenesis-enriched genes (gray-shaded sets in (**a**) and (**b**)) shows that sperm are enriched in ion binding and kinase/phosphatase activities. **(d)** GO analysis of the sperm transcriptome shows that sperm are enriched in ion binding and phosphatase activities. **(e)** GO analysis of the sperm proteome shows that sperm are enriched in ion binding and kinase/phosphatase activities. **indicates *P* < 0.01 (hypergeometric probability test).

By microarray analysis, Reinke *et al.* identified hundreds of hermaphrodite and male germline genes; the hermaphrodite germline genes were further classified as sperm-enriched, oocyte-enriched and germline-intrinsic groups [28]. These gene sets were compared with our sperm transcriptome data. The comparison showed that sperm transcriptome overlaps with 77.8% and 77.2% of the hermaphrodite and male spermatogenesis gene sets, respectively; in contrast, this sperm transcriptome covers only 18.2% and 28% of the oogenesis-enriched and germline-intrinsic gene sets, respectively (Figure 5a,b). This result shows a consistency of our sperm transcriptome data with previous microarray analysis. We were particularly interested in the hermaphrodite and male sperm genes identified in both studies (grey-shaded parts in Figure 5a,b). Gene Ontology (GO) analysis of the two sperm gene sets showed that sperm significantly enrich in kinase/phosphatase (KP) activities and ion binding activity (hypergeometric probability test, *P* < 0.01) (Figure 5c). KP has been linked to sperm function in *C. elegans*[28,29,36]; however, enrichment of the genes encoding ion binding proteins was not reported previously. Because Cl^-^, Na^+^, K^+^ and Ca^2+^ channels were implicated to modulate sperm function [37-41], ion binding proteins may play crucial roles in post-translational regulation in sperm. We also performed GO analysis of the whole sperm transcriptome, and the result resembles the above GO analysis: sperm are significantly enriched in phosphatase and ion binding activities (*P* < 0.01) (Figure 5d). Additionally, GO analysis of the sperm proteome confirms that KPs and ion binding proteins are three most enriched classes in sperm (Figure 5e). In contrast to KPs and ion binding activities, transcription factor activity and signal transducer activity are significantly low in sperm (*P* < 0.01) (Figure 5d), verifying the transcriptionally and translationally quiescent nature of sperm.

**Figure 6:**
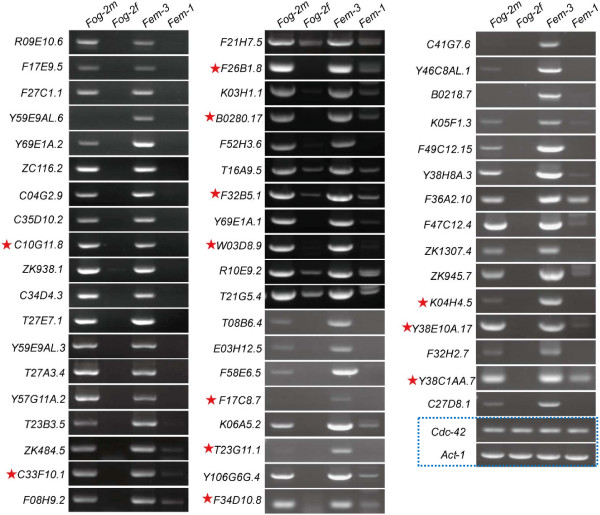
**Identification of novel sperm-specific/enriched transcripts.** RT-PCR analysis identified 53 genes that are highly expressed in males (*fog-2♂* and *fem-3*) compared with in females (*fog-2♀* and *fem-1*). Most of these genes were identified previously as spermatogenesis-enriched [28]; however, 12 genes (indicated by the red asterisks) are newly identified as being sperm-specific/enriched. The *Cdc-42* and *Act-1* genes were used as controls.

We also reasoned the transcriptome dataset has deeper depth than previous microarray data, thus we employed RT-PCR and used the germ cell mutants *fem-1*, *fem-3* and *fog-2* to screen for novel sperm specific/enriched genes. The expression of the genes with RPKM above 2.5 (reads counts >100) in sperm transcriptome was surveyed in these mutants (MSP and some well-known sperm-specific genes were excluded in this analysis), and 53 genes that represent 56% of the genes investigated were shown to be sperm-specific/enriched (Figure 6). Of these 53 genes, 12 were not defined to be spermatogenesis-enriched in previous microarray analysis (indicated by asterisks in Figure 6). Expression of the 53 genes is mostly detectable in both males (*fem-3* and *fog-2♂*) and hermaphrodites (strain N2)(Additional file 1: Figure S2), suggesting that they play common roles in the sperm of both sexes. In this screen, we identified sperm-specific/enriched genes in so high efficiency, demonstrating the sperm transcriptome data serves as a valuable resource for studying spermatogenesis.

### Sperm gene/protein abundance exhibits a skewed distribution

**Figure 7:**
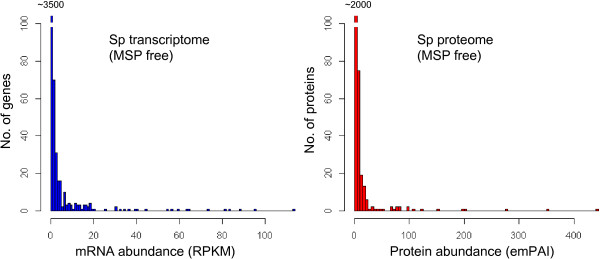
**Sperm transcriptome and proteome show skewed abundance.** Histograms showing the distributions of sperm gene (left panel) and protein (right panel) abundances.

When examining sperm mRNA/protein abundance (MSPs were excluded), we found that the majority of sperm mRNAs/proteins have very low abundance, whereas a small set of mRNAs/proteins are hugely enriched in sperm (Figure 7). We should note that biased expression for male-enriched genes has been observed in zebrafish [42], fly [43], and recently in *C. elegans*[44]. These results indicate that sperm may only rely on a small number of proteins to complete their post-translational regulations, *e.g.*, signalling cascades regulating sperm activation, motility and interaction with oocyte.

### RNAi analysis of sperm enriched genes

**Figure 8:**
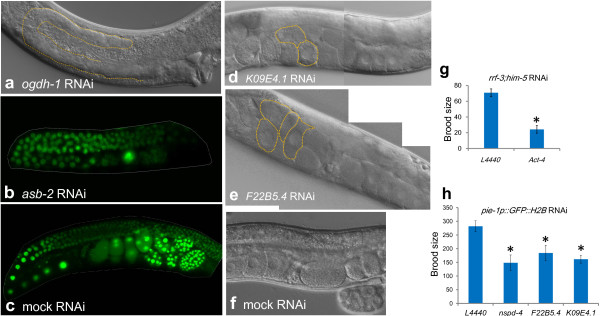
**RNAi analysis of sperm genes. (a)** Germline of the *ogdh-1* RNAi worm. Yellow dashed line marks its immature germline. **(b)***asb-2* RNAi worm showed retarded growth of the germline (~60 h after egg stage). **(c)** Mock RNAi control (the same stage as (**b**)). **(d)** and **(e)** show the germlines of *F22B5.4* and *K09E4.1* RNAi worms. Yellow dashed lines mark the unfertilized oocytes. **(f)** Mock RNAi control. **(g)** and **(h)** show the brood sizes of the *act-4*, *nspd-4*, *F22B5.4*, and *K09E4.1* RNAi worms are significantly reduced as compared with the control in which empty vector L4440 was used. *indicates *P* < 0.05 (Student’s *t*-test).

To explore the functions of sperm genes, we firstly tested the RNAi of 85 genes that have read counts above 100 using the *rrf-3;him-5* strain. Most of the RNAi-treated hermaphrodites had normal fertility. To examine the fertility of the RNAi males, we crossed them with *spe-8;dyp-4* hermaphrodites. The crossing progenies were also normal (without significant numbers of dumpy worms being observed, as compared with the control), suggesting that the sperm of these males had no defect. We identified only one gene, *act-4*, whose knockdown leads to a significantly reduced brood size (*t*-test, *P* < 0.001), as shown in Figure 8g. *Act-4* was previously shown to be expressed in spermatheca [45]. This actin is possibly involved in the contraction of spermatheca.

We further tested the RNAi of 486 genes that have read counts above 20 using the strain *pie-1::gfp::H2B* at 25°C (25°C is a challenging condition for sperm fertility, *e.g*., *fer-1*, *spe-9* and *rrf-3* all control fertility in a temperature-sensitive manner). RNAi was conducted over three generations [46]. However, our RNAi assay did not produce as many defective phenotypes as those collected from the Wormbase RNAi dataset. We describe five unreported genes that control fertility as below. First, RNAi for the gene *ogdh-1* or *asb-2* resulted in complete sterility. OGDH-1 is a mitochondrial 2-oxoglutarate dehydrogenase. *ogdh-1* RNAi worms had severe germline defects without germ cells being produced (Figure 8a). ASB-2 is the subunit of a mitochondrial ATPase. *asb-2* RNAi worms failed to produce living embryos and its germline growth was retarded when compared with the RNAi control (Figure 8b,c show the germlines at ~60 h after egg stage). The dissected germline of some *asb-2* RNAi worms evidently lacked sperm (Additional file 1: Figure S4). Moreover, RNAi for the genes *nspd-4*, *F22B5.4* and *K09E4.1* led to production of unfertilized oocytes (Figure 8d,e and Additional file 1: Figure S5). The brood sizes of the RNAi worms for these three genes were significantly reduced when compared with that of RNAi control (*t*-test, *P* < 0.01) (Figure 8h). The observation of unfertilized oocytes in the uterus of RNAi worms as well as reduced brood sizes hints that these genes are involved in spermatogenesis.

Collectively, our RNAi screen identified a few genes that regulate germline development and sperm fertility. It is worth noting that our study, consistent with prior studies, showed that downregulation of sperm genes by RNAi feeding approach is inefficient to produce defective phenotypes [28,47].

### Endo-RNAi pathway represses sperm gene expression

**Figure 9:**
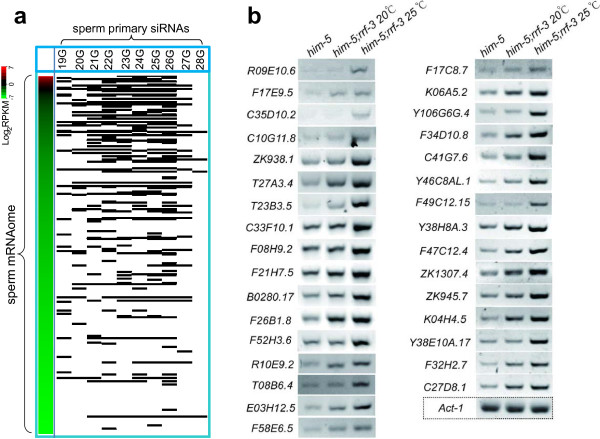
**Sperm genes are downregulated by endo-siRNA pathway. (a)** Heat-map showing the transcripts in the sperm transcriptome are targeted by antisense 19G-28G primary siRNAs. Each row represents a sperm transcript. Abundance of a sperm transcript is quantified by log_2_RPKM. The sperm transcript targeted by the corresponding siRNAs is indicated by a black line. **(b)** RT-PCR identification of 32 sperm genes that are expressed at higher levels in the *rrf-3;him-5*, as compared with the *him-5*.

Regarding to the phenomenon that RNAi of sperm genes produces fewer defective phenotypes than the corresponding mutants, we speculated it is due to that the exogenously triggered RNAi pathway could not compete over the endogenous RRF-3/ERI-1/ALG-3 RNAi pathway. The competition of these two RNAi pathways has been proposed [14]. Supporting this hypothesis, mutants lacking RRF-3 and ERI-1 have been successfully used as tools for enhanced RNAi screening. When comparing our sperm transcriptome data with the published sperm primary siRNAs data [16], we did find the sperm 19G-28G primary siRNAs have a strong correlation with sperm transcriptome; *i.e.*, for the sperm-enriched mRNAs, the corresponding antisense siRNAs are also enriched (Figure 9a, details in Additional file 7). This result further supports the previous view that the endo-siRNA pathway represses the expression of sperm genes [17].

We also examined the expressions of the 53 sperm specific/enriched genes (shown in earlier result) between *him-5* and *him-5;rrf-3* adult worms (*him-5;rrf-3* worms produces offspring at 20°C, whereas are sterile at 25°C) by RT-PCR (25 amplification cycles). Notably, of the 53 genes, 32 genes were expressed at elevated levels in the *him-5;rrf-3* as compared with *him-5* (Figure 9b). This expression elevation is particularly evident for worms grown at restrictive temperature (25°C). This result demonstrated that RRF-3, as a vital component of the endo-siRNA pathway, negatively regulates the expression of sperm genes. Together, we propose that the inefficient RNAi of sperm genes might result from the competition between exogenous RNAi and endogenous RNAi pathways during spermatogenesis (see Discussion).

## Discussion and conclusion

The sperm cell has highly condensed nucleus packed with protamines instead of histones and lacks many essential organelles, such as ribosomes and Golgi apparatus [1]. Therefore, sperm cell is devoid of transcriptional and translational activities. The long assumed view was that, at fertilization, sperm transmits solely its DNA to the oocyte, which has all the components necessary for early embryonic development. However, this notion has been changed by recent advancement of high-throughput technologies that made the unravelling of the sperm transcriptome and epigenome possible. It was discovered that sperm not only retains the histones that may play significant roles during early embryogenesis [1,5,7,48-50], but also contains complex RNA species, including mRNAs and non-coding RNAs, some of which are vital for early embryonic development [4,11]. *C. elegans* is an easily manipulated model organism, and is ideal to study sperm biology. By microarray analysis, Reinke *et al.* identified spermatogenesis, oogenesis and sex-regulated genes in *C. elegans*[28]. To improve study of spermatogenesis, here, we isolated highly pure sperm cells and obtained sperm transcriptome and proteome by high-throughput approaches.

First, sequencing sperm transcriptome showed that considerable numbers of lncRNAs are present in sperm. lncRNAs are a major group of ncRNAs whose functions are poorly understood. In *Drosophila*, some lncRNAs were thought to be involved in male-specific processes related to sexual dimorphism [34]. Recently, Nam *et al.* showed that, in *C. elegans*, many lncRNAs are associated with processes such as dauer formation, male identity, sperm formation, and interaction with sperm-specific mRNAs [35]. In our small-scale screen, eight novel lncRNAs were isolated, one of which was specifically expressed in males.

We showed that sperm gene sets are significantly enriched in not only kinase/phosphatase genes as previously reported [28,29], but also genes encoding ion binding proteins. Sperm development is regulated posttranslationally. The Cl^-^, Na^+^, K^+^ and Ca^2+^ ion channels have been implicated to respond to extracellular signals and mediate sperm activation [36-41]. Therefore, our identification of the sperm genes encoding ion binding proteins will help uncover the intracellular ion regulated pathways associated with sperm function.

We showed that sperm genes or proteins have skewed abundance, *i.e.*, a small set of genes/proteins are greatly enriched in sperm. This supports previous observations that male-enriched genes have biased expressions than female-enriched genes in zebrafish [42], fly [43] and *C. elegans*[44]. This result may suggest that compared with somatic cells, sperm only require a small number of proteins to complete their post-translational regulations, *e.g.*, signalling factors for sperm activation, MSP dynamics and interaction with oocyte.

By RNAi of hundreds of sperm-enriched genes, we identified a few genes, whose knockdown leads to production of unfertilized oocytes and significantly reduced brood size. It is worth noting that previous study showed that RNAi of sperm genes is inefficient for producing defective phenotypes [28,43]. To our knowledge, the mechanism behind this phenomenon has been unclear. It could be caused by the low penetrance of RNAi to germline tissue. Because endo-RNAi pathway strongly acts during spermatogenesis [15,17], here, we presume that this phenomenon might result from the competition between endogenous and exogenous RNAi pathways. The endogenous RNAi pathway may compete over the exogenous RNAi pathway, leading to the failure of RNAi of sperm genes.

## Methods

### *C. elegans* strains and growth conditions

The following strains were used in this study: wild-type Bristol strain N2, *spe-8(hc40)I, rrf-3(pk1426)II*, *dpy-4(e1166)IV*, *fem-1(hc17)IV*, *fem-3(q23)IV, fog-2(q71)V, him-5(e1490)V, pie-1p::gfp::H2B.* All the strains were maintained at 16°C or 20°C. The strains *fem-1* and *fem-3* are temperature-sensitive. At 25°C, *fem-1* only produces oocytes, while *fem-3* overproduces sperm.

### Large-scale culture of *C. elegans* and purification of mature sperm

Large-scale culture of *C. elegans* strain *him-5* and purification of mature sperm cells were performed using a modified protocol based on published methods [51-53] (detailed protocols in Additional file 1).

### cDNA synthesis and 454 pyrosequencing

The total RNA of *C. elegans* sperm was prepared using a *mir*Vana miRNA Isolation kit (Invitrogen, Carlsbad, CA, USA), according to the manufacturer’s instructions. Trace genomic DNA was removed using RNase-free DNase I (Promega, Madison, WI, USA). The DNase I-treated RNA was reverse transcribed to cDNA using a SMARTer PCR cDNA Synthesis kit (Clontech, Mountain View, CA, USA) and then amplified for 20 cycles using an Advantage 2 PCR kit (Clontech). Amplified cDNA was purified using a QIAquick PCR Purification Kit (Qiagen, Hilden, Germany) and 0.5 μg of the cDNA was subjected to 454 pyrosequencing using a GS FLX Titanium General Library Preparation Kit (Roche 454 Company, CT, USA).

### Sperm protein preparation and mass spectrometry analysis

*C. elegans* sperm was suspended in PBS solution, disrupted by sonication for 8 s (repeated three times at intervals of 8 s), mixed with SDS loading buffer and heated for 10 min. After centrifugation, the supernatant was loaded on a 12% SDS-PAGE gel for separation. The resulting gel was silver stained using a Fast Silver Stain kit (Beyotime, Shanghai, China). The major sperm protein (MSP) band was removed prior to tryptic in-gel digestion, according to standard protocols. The tryptic peptides were extracted using 60% acetonitrile in 0.1% formic acid, dried in vacuum and then resuspended in 0.1% formaic acid for mass spectrometry analysis. LC-MS/MS analysis was performed in a LTQ-orbitrap XL (ThermoFinnigan, San Jose, CA) coupled online with an Eksgent Nano 2D LC system. Peptides mixture was first loaded on a trap column (300SB-C18, 5 × 0.3 mm, 5 μm particle) (Agilent Technologies, Santa Clara, CA), and then analysed using a self-packed capillary C18 column (75 μm i.d. × 150 mm, 3 μm particle, C18 resin), and eluted with a gradient of 4-35% of Buffer B (0.5% formic acid in acetonitrile) in Buffer A (0.5% formic acid in water) at a flow rate of ~300 nl/min for 120 min. Data-dependent scanning was incorporated to select the 10 most abundant ions from a full-scan mass spectrum (mass range 200–1800 Da) for fragmentation by collision-induced dissociation.

### Mass spectrometry data processing

MS data were analyzed using Mascot (Matrix Science, version 2.3.02) against the *C. elegans* protein dataset (release WS229) with the following parameters: only tryptic peptides with up to two missed cleavage sites were allowed; 20 ppm mass tolerances for MS; and 0.8 Da for MS/MS fragment ions. Peptides with Mascot Percolator posterior error probability values lower than 0.05 were considered to be potential candidates. Decoy database searches in Mascot revealed a false positive rate of 1.25% at the peptide level. Proteins with ≥2 unique peptides were accepted as matches.

### Bioinformatic analyses

The raw 454 sequencing reads were filtered by trimming off the adaptor sequences and removing short reads (<50 bp). Repeat sequences were masked, and tRNA, rRNA, snoRNA and snRNA sequences (WS228) were filtered before reads assembling using Newbler, version 2.3 (Roche). For sequence assembly, the *C. elegans* coding transcript data (WS228) was used as a reference; the unassembled reads were further assembled using the *C. elegans* genome as a reference. For the assembly we used the Newbler default parameters (overlap length >40 bp, identities >90%). Sequences in the non-coding portion were evaluated for coding potential using CPAT (http://lilab.research.bcm.edu/cpat/index.php). IGV genome browser (http://www.broadinstitute.org/igv/) was used to view reads assembling information for novel non-coding RNA transcripts. Sperm transcriptome was functionally annotated using the GO Slimmer tool (http://amigo.geneontology.org/cgi-bin/amigo/slimmer?session_id). Sperm primary siRNAs data described previously [16] were downloaded from the NCBI GEO database (http://www.ncbi.nlm.nih.gov/geo/). These siRNAs datasets were compared to our sperm transcriptome data using BLAST, and the siRNAs with a perfect antisense match were retained as the siRNAs targeting sperm genes. *C. elegans* polyadenylated lncRNA data was previously described [35]. *C. elegans* genome data, cds transcript dataset and annotation data were all downloaded from Wormbase (ftp://ftp.wormbase.org/pub/wormbase/species/c_elegans/).

### 3′-rapid amplification of cDNA ends (3′RACE)

DNase I-treated sperm RNA was reverse transcribed into cDNA following the 3′RACE protocol in the FirstChoice RLM-RACE kit (Invitrogen). Nested PCR was conducted using the High Fidelity PCR SuperMix (TransGen, Beijing, China) and the amplified product was cloned into the *pEASY*-Blunt vector (TransGen) before sequencing to identify its 3′ sequence.

### Reverse transcription PCR (RT-PCR)

The N2, *fem-1(lf)*, *fem-3(gf)* and *fog-2(lf)* strains were synchronized and the adults were collected and frozen as starting materials. Total RNAs from these strains were isolated using a *mir*Vana miRNA Isolation kit (Invitrogen). Total RNA (2 μg) was reverse transcribed into cDNA using the SuperScript III First-Strand Synthesis System (Invitrogen). The cDNA product was diluted 10-fold and used as the template for RT-PCR. The *Act-1* and *Cdc-42* genes were used as controls.

### Microscopic analysis

The RNAi treated *pie-1p::gfp::H2B* strain were anesthetized and mounted on agarose pads for visualization using a differential interference contrast (DIC) microscope (Zeiss, Axio Imager M2) as well as a confocal microscope (Zeiss LSM 510 Meta). The gonads of *asb-2*RNAi worms were dissected, and then fixed by cold methanol method (http://www.wormbook.org/toc_wormmethods.html), followed by DAPI staining and microscopic visualization.

### RNA interference screen

The RNAi screen was performed as previously described [54,55]. Briefly, the strain *rrf-3;him-5* or *pie-1p::gfp::H2B* was fed with the RNAi bacteria obtained from the Ahringer RNAi library [56]. Bacteria carrying the empty vector (L4440) were used as the control. The bacteria clones that affect fertility were sequenced to verify the gene sequences.

### Brood size assay

The L4 stage hermaphrodites of the RNAi worms were individually picked onto plates, and transferred to new plates daily until no eggs were laid. Brood size was determined by counting the worms on all plates. 50 replications were performed. Worms fed with bacteria carrying L4440 were used as the control.

## Availability of supporting data

The raw sperm transcriptome sequencing data has been deposited in NCBI SRA database with the accession number of SRA056374. Sperm mRNA-seq assembled data and sperm proteomics data can be downloaded from http://159.226.118.206/miaolab/C.elegans%20data.htm.

## Competing interests

The authors declare that they have no competing interests.

## Authors’ contributions

XM and LM designed the study and wrote the manuscript. XM prepared the samples. CL performed 454 pyrosequencing. PX carried out proteomics analysis. XM and YiZ carried out bioinformatic analysis. XM performed RT-PCR analysis. XM and YaZ performed RNAi screening. FY, SC helped to coordinate the experiments. All authors read and approved the final manuscript.

## Supplementary Material

Additional file 1: Table S1 Summary of sperm transcriptome sequencing. **Table S2.** Somatic marker genes used to evaluate contamination levels in the sperm transcriptome. **Figure S1.** Length distribution of the sequencing reads in the sperm mRNAome. Most of the reads are longer than 400 bp suggesting high quality sequencing. Purple line indicates average length. **Figure S2.** RT-PCR expression analysis of 51 genes in the N2, *fem-3* and *fem-1* strains. These genes have biased expressions in the male (*fem-3*) and hermaphrodite (N2) compared with their expressions in the female (*fem-1*), suggesting that they are sperm-specific/enriched. The genes *Cdc-42* and *Act-1* were used as controls. **Figure S3.** DAPI staining of the gonad of the RNAi control and one *asb-2* RNAi worm. White arrows indicate that sperm are present in the mock RNAi control; yellow arrow indicates the absence of sperm in one *asb-2* RNAi worm. **Figure S4.***F22B5.4* RNAi worm produces unfertilized oocytes. Arrows indicate the unfertilized oocytes from the cracked body. Supplementary methods. Large-scale culture of *C. elegans* and purification of mature sperms. Click here for file

Additional file 2 **Assemblies mapped to *C. elegans *** **CDS transcriptome.** Sequences available from http://www.ibp.cas.cn/MiaolLab/index.html. Click here for file

Additional file 3 **Assemblies mapped to *C. elegans *** **genome, but unmapped to CDS transcriptome.** Sequences available from http://159.226.118.206/miaolab/index.htm. Click here for file

Additional file 4 List of sperm genes with RPKM above 3.27e-3. Click here for file

Additional file 5 **Genes/Proteins found in *C. elegans *** **sperm transcriptome and proteome.** Click here for file

Additional file 6 Sequences of eight novel lncRNAs confirmed by 3′RACE. Click here for file

Additional file 7 ***C. elegans *** **sperm genes targeted primary siRNAs.** Click here for file
