# Supplementary material for: Characterisation of Caenorhabditis elegans sperm transcriptome and proteome
Source: BMC Genomics. 2014 Feb 28;15:168. doi: 10.1186/1471-2164-15-168 (PMC4028957; doi:10.1186/1471-2164-15-168)
Supplement: Additional file 1: Table S1 — Summary of sperm transcriptome sequencing. Table S2. Somatic marker genes used to evaluate contamination levels in the sperm transcriptome. Figure S1. Length distribution of the sequencing reads in the sperm mRNAome. Most of the reads are longer than 400 bp suggesting high quality sequencing. Purple line indicates average length. Figure S2. RT-PCR expression analysis of 51 genes in the N2, fem-3 and fem-1 strains. These genes have biased expressions in the male (fem-3) and hermaphrodite (N2) compared with their expressions in the female (fem-1), suggesting that they are sperm-specific/enriched. The genes Cdc-42 and Act-1 were used as controls. Figure S3. DAPI staining of the gonad of the RNAi control and one asb-2 RNAi worm. White arrows indicate that sperm are present in the mock RNAi control; yellow arrow indicates the absence of sperm in one asb-2 RNAi worm. Figure S4.F22B5.4 RNAi worm produces unfertilized oocytes. Arrows indicate the unfertilized oocytes from the cracked body. Supplementary methods. Large-scale culture of C. elegans and purification of mature sperms. [file 1471-2164-15-168-S1.PDF]

SUPPLEMENTAL TABLES

TABLE S1. Summary of sperm mRNAome sequencing

	No. of sequences	Average length (bp)
**HQ sequencing reads**	367,638	315
**Assembled contigs**	10,525	508
**Singletons**	10,325	355

HQ: high quality.

TABLE S2. Somatic marker genes used to evaluate contamination levels in the sperm mRNAome.

Locus	Gene ID	Expression	No. of reads
clh-4	*T06F4.2a;T06F4.2b*	excretory cells	0
dpy-7	*F46C8.6*	hypodermis	0
bli-3	*F56C11.1*	hypodermis	0
wrt-2	*F52E4.6*	seam cells and hypodermis	2
ges-1	*R12A1.4*	intestine	1
elt-2	*C33D3.1*	intestine	0
pept-1	*K04E7.2*	intestine	0
hlh-17	*F38C2.2*	YA cephalic sheath	1
myo-2	*T18D3.4*	pharyngeal muscle	2
myo-3	*K12F2.1*	body muscle	3
pept-3	*F56F4.5*	AVE neurons	0
rig-3	*C53B7.1*	AVA neurons	0
unc-4	*F26C11.2*	A-class neurons	0
dat-1	*T23G5.5*	dopaminergic neurons	0
rgef-1	*F25B3.3*	all neurons	0

Figure S1

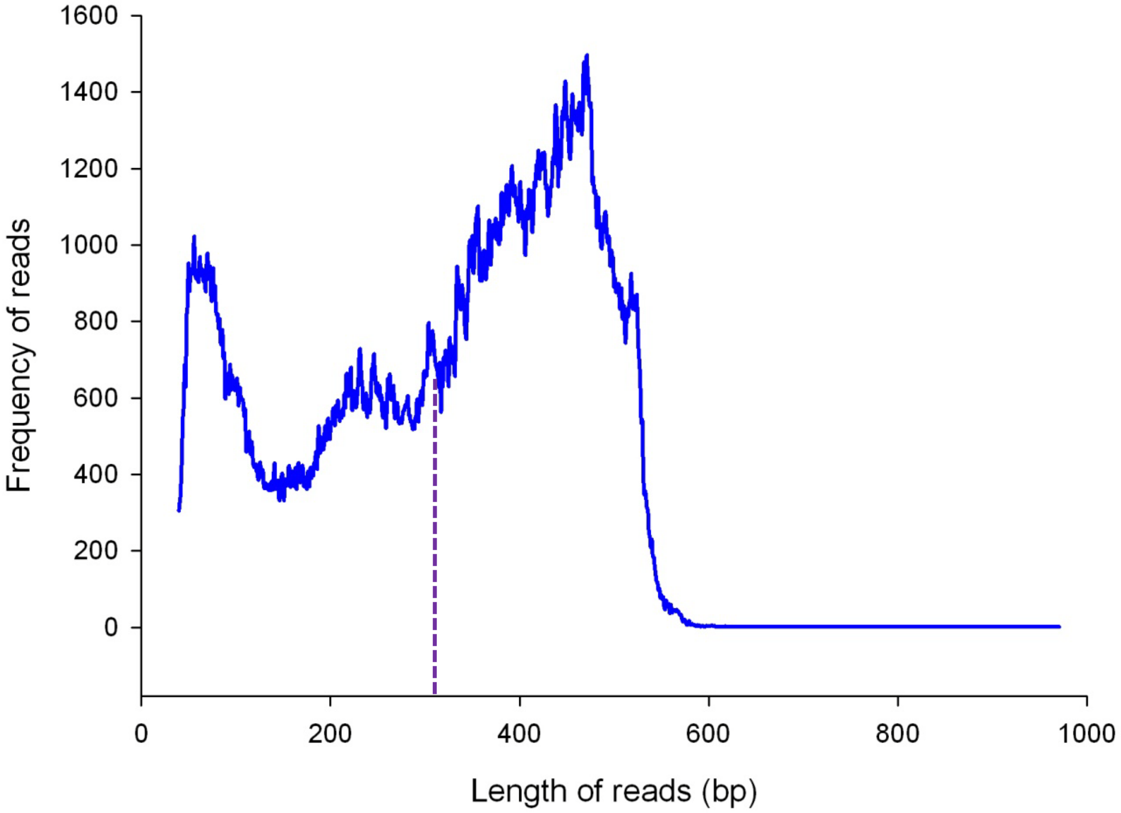

1600
1400
1200
1000
800
600
400
200
0
Frequency of reads
0
200
400
600
800
1000
Length of reads (bp)

Figure S2

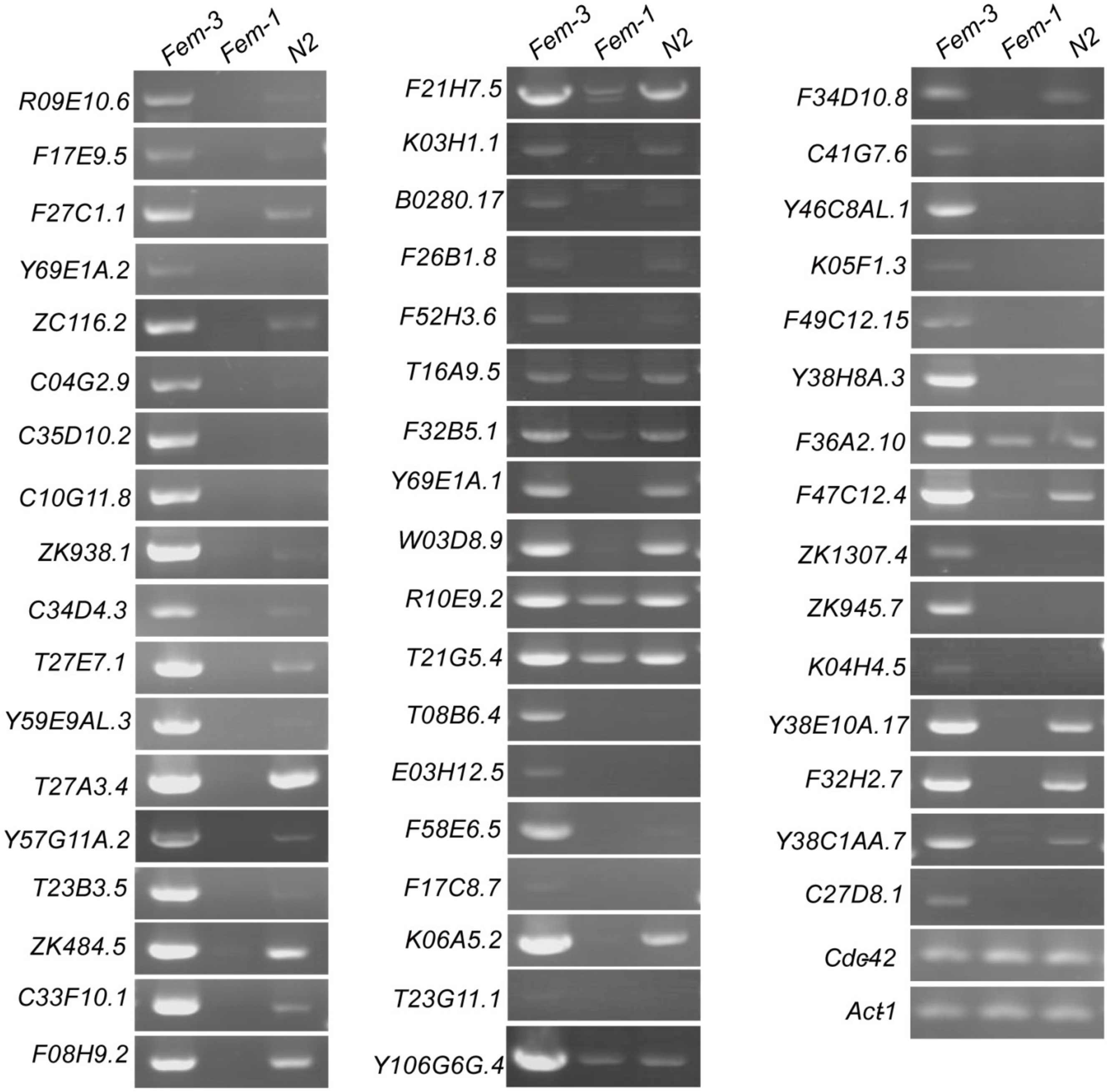
Fem-3
Fem-1
N2
R09E10.6
F17E9.5
F27C1.1
Y69E1A.2
ZC116.2
C04G2.9
C35D10.2
C10G11.8
ZK938.1
C34D4.3
T27E7.1
Y59E9AL.3
T27A3.4
Y57G11A.2
T23B3.5
ZK484.5
C33F10.1
F08H9.2
F21H7.5
K03H1.1
B0280.17
F26B1.8
F52H3.6
T16A9.5
F32B5.1
Y69E1A.1
W03D8.9
R10E9.2
T21G5.4
T08B6.4
E03H12.5
F58E6.5
F17C8.7
K06A5.2
T23G11.1
Y106G6G.4
F34D10.8
C41G7.6
Y46C8AL.1
K05F1.3
F49C12.15
Y38H8A.3
F36A2.10
F47C12.4
ZK1307.4
ZK945.7
K04H4.5
Y38E10A.17
F32H2.7
Y38C1AA.7
C27D8.1
Cdc42
Act1

Figure S3

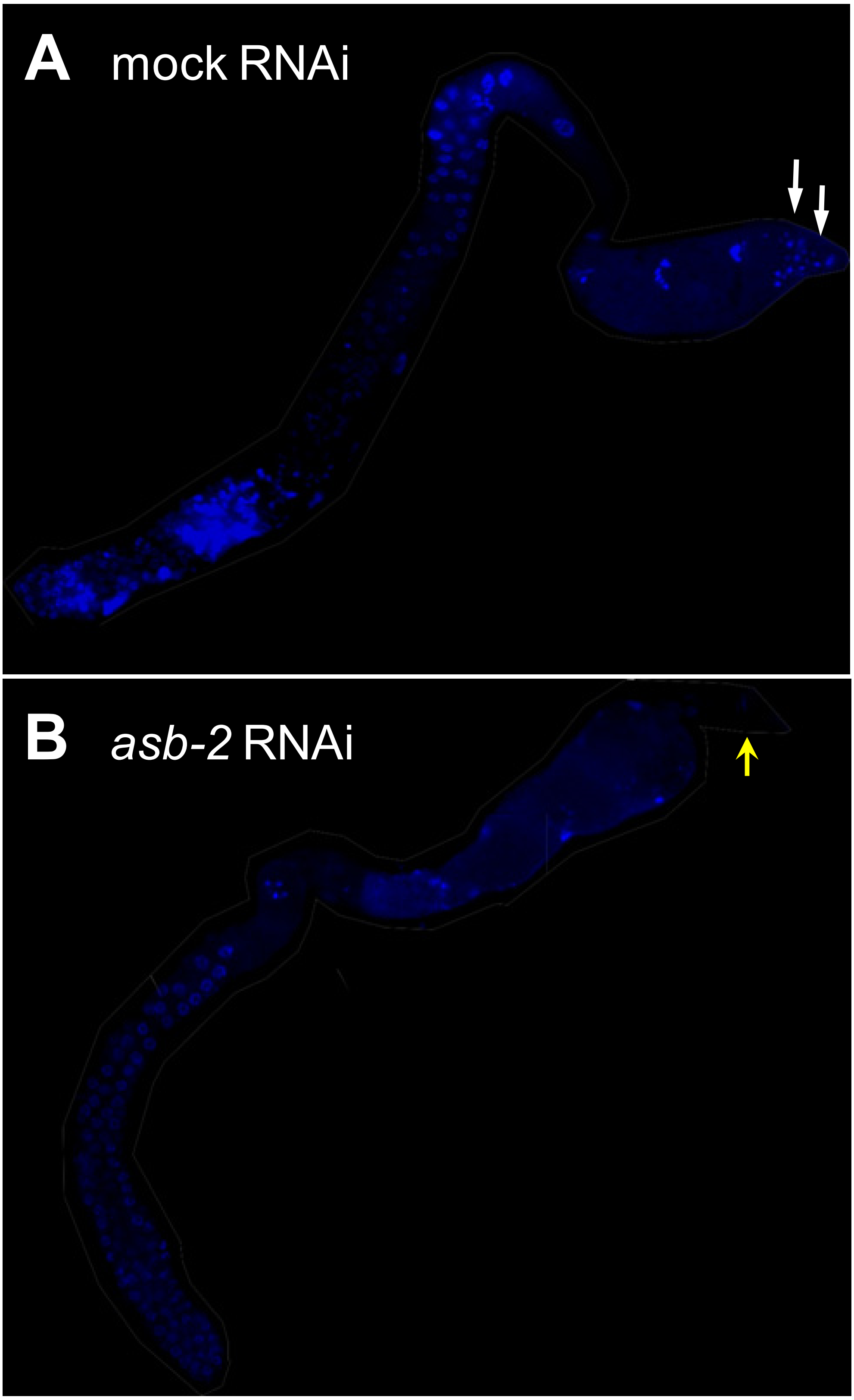

Figure S4

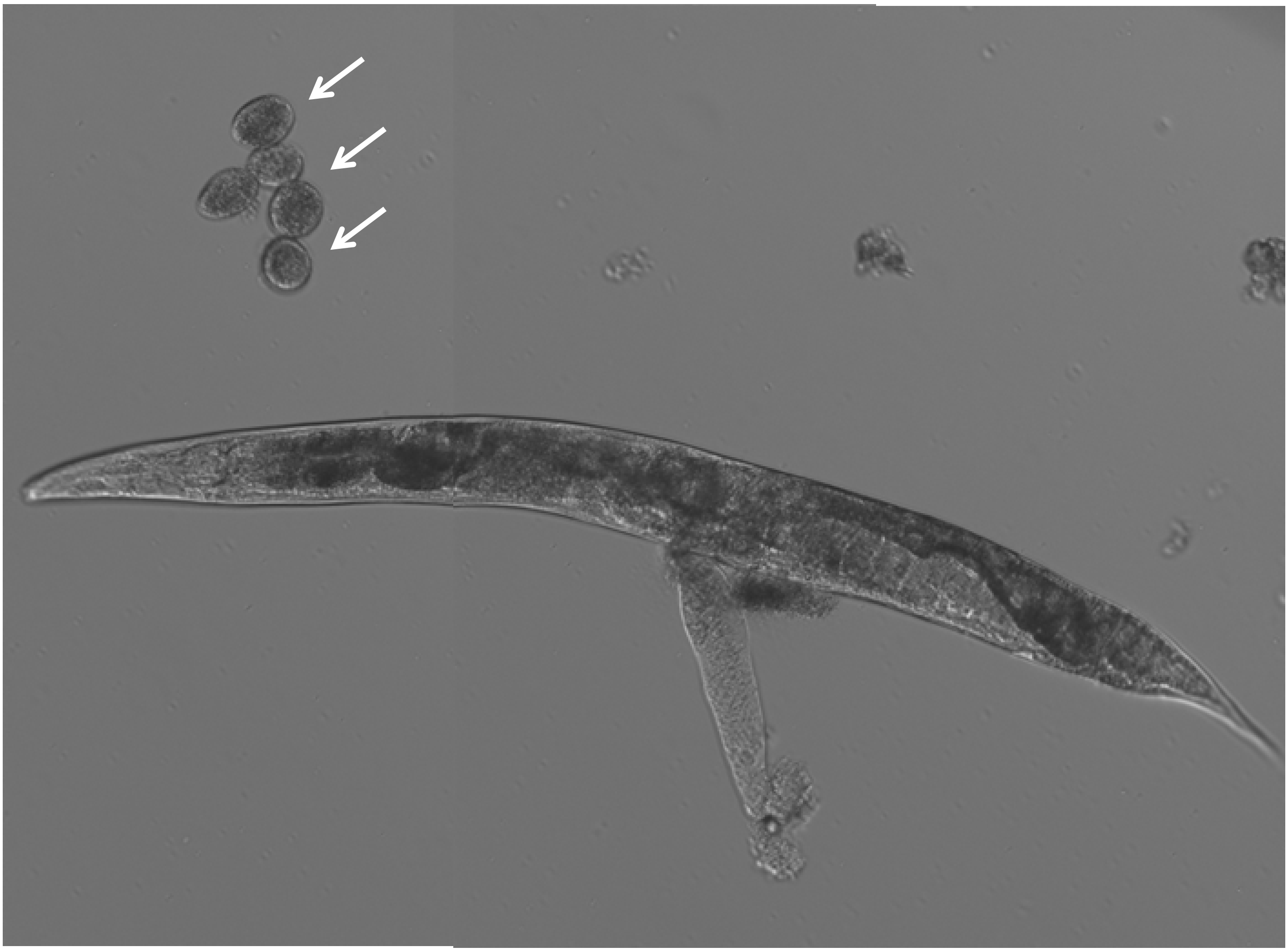

SUPPLEMENTAL FIGURE LEGENDS

FIGURE S1. Length distribution of the sequencing reads in the sperm mRNAome.

Most of the reads are longer than 400 bp suggesting high quality sequencing. Purple line indicates average length.

FIGURE S2. RT-PCR expression analysis of 51 genes in the N2, *fem-3* and *fem-1* strains.

These genes have biased expressions in the male (*fem-3*) and hermaphrodite (N2) compared with their expressions in the female (*fem-1*), suggesting that they are sperm-specific/enriched. The genes *Cdc-42* and *Act-1* were used as controls.

FIGURE S3. DAPI staining of the gonad of the RNAi control and one *asb-2* RNAi worm.

White arrows indicate that sperm are present in the mock RNAi control; yellow arrow indicates the absence of sperm in one *asb-2* RNAi worm.

FIGURE S4. *F22B5.4* RNAi worm produces unfertilized oocytes.

Arrows indicate the unfertilized oocytes from the cracked body.

SUPPLEMENTAL METHOD

Large-scale culture of *C. elegans* and purification of mature sperm

Large-scale culture of *C. elegans* strain *him-5* was performed as following protocols. Strain *him-5* was grown on 20 nematode growth media (NGM) plates at 20°C until bacteria lawns (*E. coli* OP50) became clear. The worms were rinsed and resuspended in M9 buffer. 1/2 volume of 60% sucrose was then added, followed by centrifugation to separate the worms from the bacteria. Worms were recovered and synchronized using a bleach/NaOH mixture (5% sodium hypochlorite, 0.5 M NaOH). Eggs were washed and grown for 16–24 hrs in M9 buffer with constant shaking. The L1 worms were pelleted, and grown in M9 supplied with bacteria (*E. coli* X1666), until they reached the adult stage. Adults were collected by sucrose flotation, resuspended in M9 and filtered through a 35 μm Nytex nylon mesh. The hermaphrodites were retained on the top of the mesh. Worms at the bottom of the mesh were further filtered through a 20 μm nylon mesh. Males on the top of the 20 μm mesh were rinsed with M9. The males were settled for 10 min and resuspended in PSM buffer, before being transferred to a glass Petri dish and chopped for 5 min. The chopped worms were filtered through a 10 μm mesh (repeated three times). The filtrate was layered on 10% Percoll and centrifuged at 800 ×*g* for 10 min. The pellet (purified sperm cells) was resuspended in SM buffer and subjected to microscopic examination. The purified sperm cells were immediately frozen at -80°C.
