# Supplementary material for: Characterisation of Caenorhabditis elegans sperm transcriptome and proteome
Source: BMC Genomics. 2014 Feb 28;15:168. doi: 10.1186/1471-2164-15-168 (PMC4028957; doi:10.1186/1471-2164-15-168)
Supplement: Additional file 6 — Sequences of eight novel lncRNAs confirmed by 3′RACE. [file 1471-2164-15-168-S6.DOCX]

**Additional file 6**

**Sequences of eight novel lncRNAs confirmed by 3’RACE**

>Spnc1

ATGCCCATCCTGGTATGCTGTTGCTCCTTGAGCATTATGATTGCGTAAAACATGTCCAACAGAGCTTGTATTGCAAGTCAGAAAACCACATTTAACTCACCTTTTCAGTTCTCGTATTCGAATCACAACGACAATGGAATCCTATCAATAAAGAACAATCAATAAATTTTCTGTTGGTATCGTTTTTTACAGTTAGTGTTGGAATATAGAATTTCACGTTTGACCCGAGTTTTTTTTTATAGTTCACGAAATATATTATTTCAATTCAATACTTTATTCAATTAAAAACACAAAAAAAAAAAAA

>Spnc2

CGGAAATTCACTCGGATTGCTGTCTTTTTTTTCTCGCGTTGAATAATTAGAATCATATGATTCTCTTAATTTTTCAATAGAGCTTCCAATTTGTGTGATGAAAATGTATTATTTCAAACTATGTATTTGTATTTTTCTTTTGAAAAGAAAATAGTTTGCAACGCCAATAACAATTTTATCTACCGAAATTTGTCCTAAAATTAGCACATATTTTTTTGCCTGGTAGTGCCTGATAATTAAAAAACAAATTTCTCGATTATAATTCTTGCTTTTTCAAATTTCAGTTCCCCAAAAAAAAAAAAA

>Spnc3

TaCAACTGTATTTACAACCTCAGGGCGACGACGACTCCAGTGGCTCTATTGTGAGTAATTCATTCATTCATGTTTTCGTGGGCGACTGGTGGTTGCCCGTTTTCTTATTCTAACTAATATTCTATCAAACTATTTTATATTCTTCTCGAACACCTGTGTTTCTGAAACATTTGGTAAATCACGTTTTCTTTTATTTATAGGTGGAGTAGCGCCAGGGTTGTTGCAAAAAATGTTCGAACAAAAAAAAAAAAA

>Spnc4

TGTGTAGCTGCACTATCAATACAGGATTTCGGCAGTTCACTTTTCGAGCTGGTCTTCAGAGAAGTGTCTTATATAGAAAAATGTTGAGAGCCTTTTTTATTGTCGTTACTATTTTCTGTCATTACAATACAACAGGCCGACAACTTGGAACACTATTATTCAGTTTTTGTCATATGTTTTGTAGTGACTCAAAAAAAAAAAAA

>Spnc5

GTCTTATTACAGTTTGGTCAACAAAATGTGCGGTACTTTTTCCCTAAAAAAAATGTGACGTCAGTACGTTCTTAACCATGCGAAATCAATTGAGCAGTATGCATCTAAATTCAAACCGAAATGGGACACACTGACACCACGTTGTGGTTTCGAACACTTTCAAAAAAAAAAAAA

>Spnc6

ATGCGGGAGAAGAGACGCAGAGTTCTCAACTGATTTCGCATGGTTAAGAACCTGATGACGCCACTTTTTTGGGAAAAGAATTCCCGCTTTTTTTGTAGATCAAACCGTAATGGGACGGTCCGGCACCACGTGGAGTACCACTACTTCCAAGTGCCATACAAAAACTTGAGAGCATCCAATTTACAGGCGGTGCAATGAACATGACTGATATGGATCCTAGTAACATGACGGGAGGTCCAATGAATGGGCACTCGGCGGAAAAGTCTTCCAAAAAAAAAAAAA

>Spnc7

AGAACTACTGTAGTTTTCGGTGCGAGATATTCTGCGCGTCAAATATGTTGCGCAATACGCATCCTCAAAACGTAGTGTTCCCGTAATACGTTATTTCCAGACATTTAATTGTTTTTTAAAGAATGTTTAGAAGTGGGCACGCAGTACAGAAATTTTTTGGAAACTATAAAAAATAGTTTTGAAAAATTGAATCATTCATCCAACTCAAAAAAAAAAAAA

>Spnc8

TATCGTTGTGAATAGTCTCTAGATATTTTCCACATTTATCCAACCTAATATTTTGGTGTACTACTGCCTAGTGATCTCCCGACCGGGCCTATCTCCCATTCTCTCCATATTTCGGTTCTCTTTTTCTCTCACTCAAAAATGGTTCTCTTCTACACTTTTTTACATGTTTTTCTATTCCTTTCCCCATATTCTGGTTTCCAAATCTCAGTTTTTGCAACTCTCACGACATGTGCATTTCTTTAGAAGACTCCACACCTGTTTTTTTAAAATTAGTTCCGGTGTTACGATTGTTGAGCGTAATGTAATAAGGCAAGTGTACATACTCCCTTCCCCTTTGAAATGTGTATCATATAATGGTAACTTGTTTGTATCCTCACTCACCTTGATCCATCTCCATCTTATGCAATCTCGGTTATTTTTACGTGGTCCCTTATTCCATCCAATGTGTGAAACATTCCACTGGTTGCCACGTCAAGGTTCCAAATTCCCGAGTGAAACAAAATCATGACCTGGCAACCTGTGGTTAGTACAAAGAAGCCTCCTCTCGGAAAAACGCGGTTATTATCTTATCCCATTCTCAATTGCCTCTTCTCACCTCTTGTCTAGTCATCAAAAGCCCCCAATTGACTTACCGTACTTGTTACACATTTCTCTGTTTTATATCTGCCCTGCCTTAAAATCTCTGTTTCCATCCGTTCCCAAAACGAGTATTCTTCCGAAAAAAAAAAAAA
